# Analysis of Updates in Multiple Myeloma Treatment and Management

**DOI:** 10.33696/haematology.4.055

**Published:** 2023

**Authors:** Maria Jacqueline Nieto, Aryles Hedjar, Margaret Locke, Jessica Caro, Muhammad Wasif Saif

**Affiliations:** 1 Department of Cancer, Northwell Health Cancer Institute & Donald and Barbara Zucker School of Medicine at Hofstra, Lake Success, NY, USA; 2 Orlando Health Cancer Institute, Orlando, FL, USA

**Keywords:** Hematologic oncology, Myeloma, T-cell therapy

## Abstract

**Introduction:**

During the past two decades, new therapeutic agents have greatly improved the treatment landscape in multiple myeloma (MM). Treatments such as proteasome inhibitors, immunomodulatory agents, targeted monoclonal antibody therapy, and chimeric antigen receptor (CAR) T-cell therapy have improved outcomes with less toxicity. Advances in laboratory testing have accompanied this change, performing faster and more accurate assessments of treatment response. Despite these advances, however, disparities in MM outcomes persist.

**Objective:**

The purpose of this study was to review epidemiological trends in MM over the past two decades and to identify disparities that may impact MM identification and survival.

**Methods:**

Retrospective analysis was conducted on adult patients diagnosed with MM between the years 2000–2019 using the November 2021 Surveillance, Epidemiology, and End Results (SEER) program database. Joinpoint models were used to calculate annual percent changes (APCs) and average annual percent change (AAPC).

**Results:**

There were a total of 111,328 diagnoses of MM extracted from the SEER database. Most patients were male (55.17%) and white (76.7%). Age-adjusted rate analysis found a significantly higher incidence among black patients compared to white patients. The APC between 2000–2015 was 1.46, and the APC between 2015–2019 was −1.34. Relative survival also increased from 2000 to 2014. The 5-year cancer survival in MM also increased at an average of 1.8% for every year after diagnosis. The annual probability of MM-related mortality at the 1-year mark also decreased from 28.5% in 2000 to 16.7% in 2018.

**Conclusion:**

Novel advances in MM therapeutic agents and diagnostic testing have paved the way for significant improvements in patient survival outcomes. Disparities persist along racial lines. Further research is needed to evaluate responses to specific MM treatment in the age of newly developed targeted therapies to overcome these disparities.

## Introduction

Multiple myeloma (MM), a monoclonal plasma cell malignancy, comprises 1–2% of all cancers and about 10–20% of all hematologic malignancies [1,2]. Every year, there are nearly 35,000 new patients who develop the disease in the United States, and nearly 13,000 people who die from it [2]. Many patients initially have a condition called Monoclonal Gammopathy of Undetermined Significance (MGUS), which increases the risk of developing MM to an average of 1% per year, depending on the specific monoclonal protein [3]. Smoldering MM (SMM) increases that risk to an average of 10% per year and nearly 75% after 15 years [3]. MM has a slight male predominance and is twice as prevalent in African-Americans as opposed to Caucasians [4]. In addition, familial history increases the chance of developing MM by 2–3 fold [5]. The median age when diagnosed is about 65–70 years [6]. It is extremely rare for patients less than 30 years old to have MM, although this incidence is also increased by three times in African-Americans [3].

Multiple myeloma is characterized by specific clinical findings, noted by the acronym CRAB (Calcium elevation, Renal Dysfunction, Anemia, and Bony Lesions), due to the triggering plasma cell clone [7]. The IMWG changed the diagnostic criteria in 2014 to include biomarkers such as an increased free light chain (FLC) ratio >100 and a population of clonal plasma cells > 60% in the bone marrow, regardless of the existence of CRAB features [1].

In addition to these diagnostic changes allowing early initiation of treatment, the medications used to treat MM have drastically changed in the past 10 years. Historically, the median overall survival (OS) was 2–3 years during the 1990s [3]. Several new medications with different mechanisms of action have been recently approved, improving survival for patients with MM. Now, first-line treatment regimens include a combination of proteasome inhibitors, immunomodulatory agents, and glucocorticoids [5]. Progression-free survival (PFS) and OS have also improved with the introduction of autologous stem cell bone marrow transplantation and targeted monoclonal antibody therapy [5–7]. 5-year OS rates have now surpassed 50% [3], largely due to the above treatments, along with maintenance therapy post-transplant [5]. For patients who have relapsed/refractory disease, novel immunotherapies provide additional therapeutic targets such as treatment with chimeric antigen receptor (CAR) T-cells. Bispecific antibodies have also shown promise, with less severe adverse effects, including low-grade cytokine release syndrome, cytopenias, and infections [8,9].

These advances are both due to and create a much better understanding of this disease. Genetic and genomic studies have revealed multiple genes that direct plasma cell development and proliferation. High-risk cytogenetics such as t(4;14), t(14;16), and del(17p) are now incorporated into risk stratification systems and influence staging [10]. Quantifying the plasma cell population in the bone marrow has also been crucial to determining an accurate response [11]. Additionally, the IMWG has developed new criteria to define disease response, emphasizing the ways to assess minimal residual disease (MRD), which is based on findings in the bone marrow flow cytometry and/or next-generation sequencing (NGS), along with imaging (PET/CT) [12]. There is an important correlation between level of response and long-term outcomes, as studies have shown better PFS and OS in patients who are MRD-negative [12].

Despite these recent advancements in the treatment of MM, outcomes still vary due to differences in race, ethnicity, and other factors that can affect how patients respond. Recognizing and understanding these disparities will create further ways to improve the care and management of all individuals.

This retrospective study used age, sex, race, stage, and mortality to analyze time trends for these categories in MM. The Surveillance, Epidemiologic, and End Results (SEER) database was used from the years 2000–2019 to determine the incidence of MM over that 18-year period. This National Cancer Institute (NCI) database used 9 population-based cancer registries throughout the United States to collect cancer incidence. We used this database to identify the above prognostic factors that could impact incidence and survival in MM. Histologic code 9732/3, was identified as Plasma Cell Myeloma.

## Methodology

Data was obtained from November 2021 data submission of the Surveillance, Epidemiology, and End Results (SEER) program. Relative survival (RS) was calculated for patients with cancer diagnosed between 2000–2019 and followed through 2019 in the SEER-17 registries using the SEER*Stat software.

We used the Joinpoint Regression Model for data analysis. This model uses analysis software created by the NCI to study trends in data gathered from the SEER Program. This program describes data trend changes by connecting several different line segments at join points. Using the minimum number of join points as an initial starting point (e.g., 0 join points, which is a straight line) the software determines whether more join points will create statistical significance and need to be added to the model (up to that maximum number). This will allow the user to determine whether a perceived change in data trends is statistically significant. Calculating joinpoints uses the Bayesian Information Criterion (BIC) and Akaike Information Criterion and calculates the Log Likelihood and Converged.

The Joinpoint Survival Model is a continuation of the Cox proportional hazards model for survival [10–12]. It can be used to analyze both Relative Survival and Cause-Specific Survival.

The annual percent change (APC) and average annual percent change (AAPC) are used to sum up and compare the rates of change that vary over a select period of multiple myeloma incidence. In general, the rate of change rises over the given period if the lower confidence limit of the AAPC is positive, or it decreases if the upper confidence limit of the AAPC is negative.

## Results

**Figure 1.**
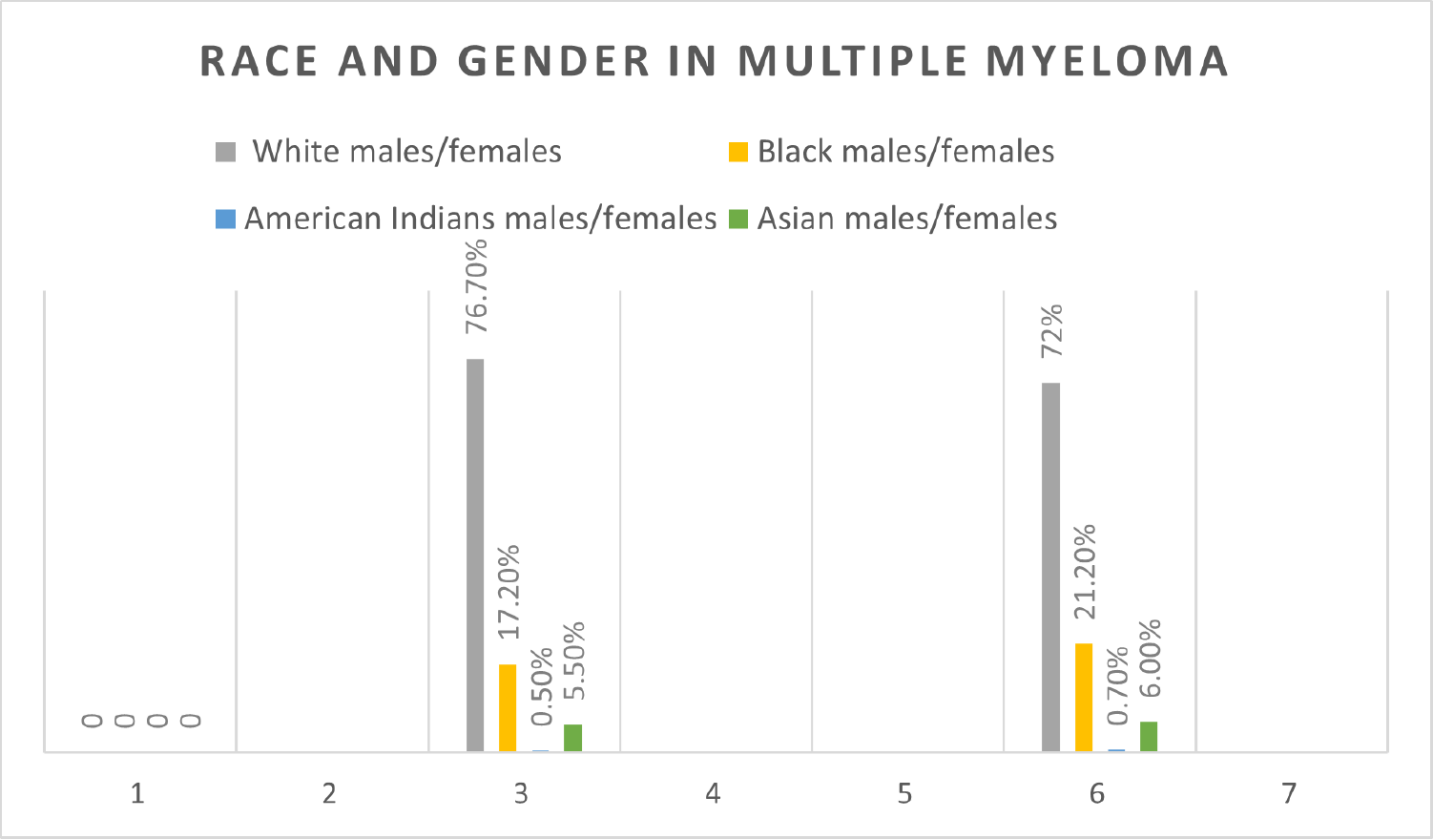
Race and gender in MM.

The characteristics of MM patients aged 30 and older as obtained from the SEER database from 2000–2019, are summarized in Table 1. The incidence of MM was greater among males (55.17%) compared to females (44.82%). Out of those males, the percentage of white patients was higher at 76.7% compared to other races, as shown in Table 2. The percentage of females who were black was higher at 21.20% compared to the percentage of males who were black (17.20%) (Figure 1).

**Figure 2.**
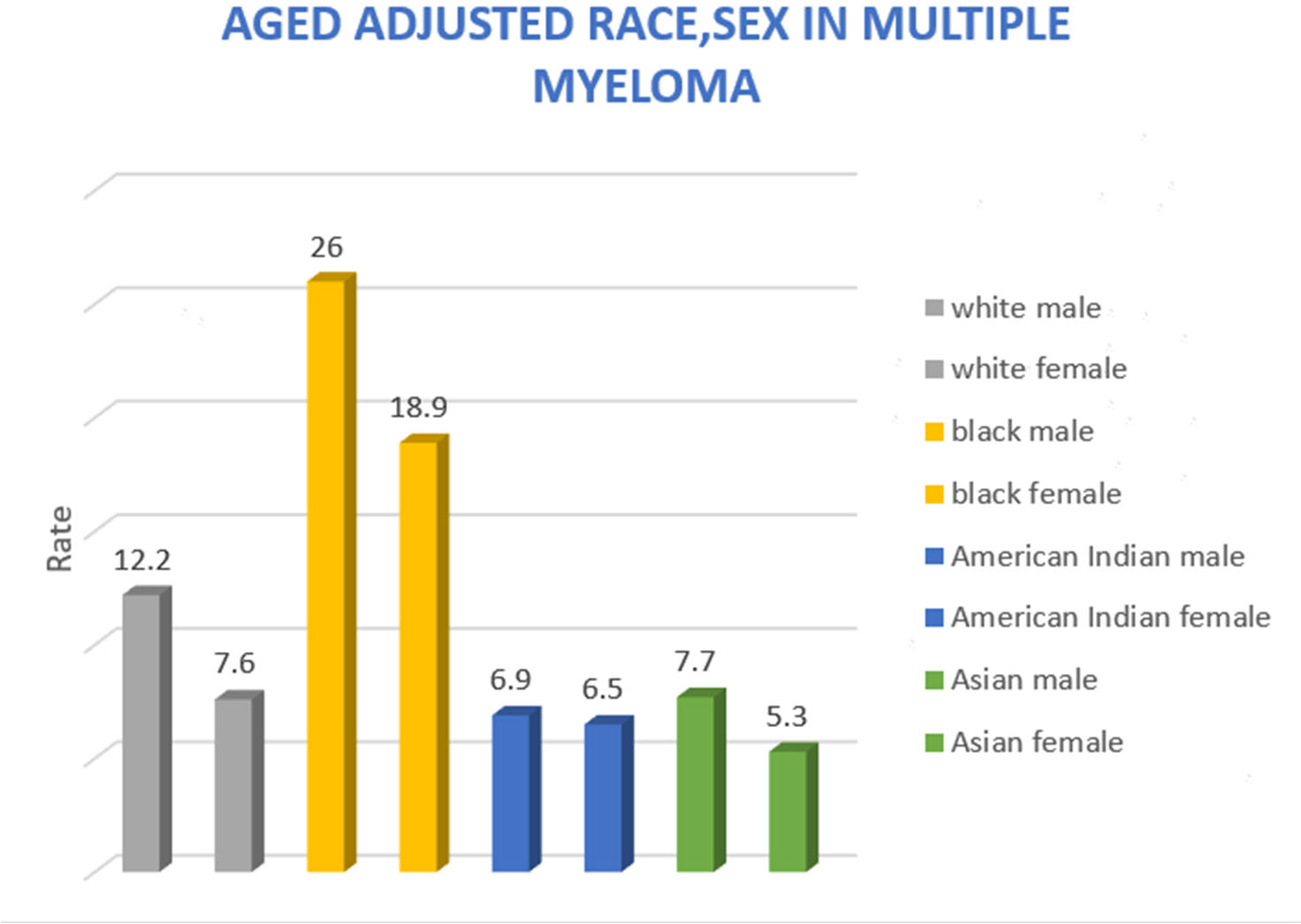
Age adjusted race and gender in MM.

The age-adjusted rate analysis for sex and race in MM is described in Figure 2. The age-adjusted rate for black males was 26, more than twice that for white males (12.2). For black females, the rate was 18.9, compared to white females (7.9).

**Figure 3.**
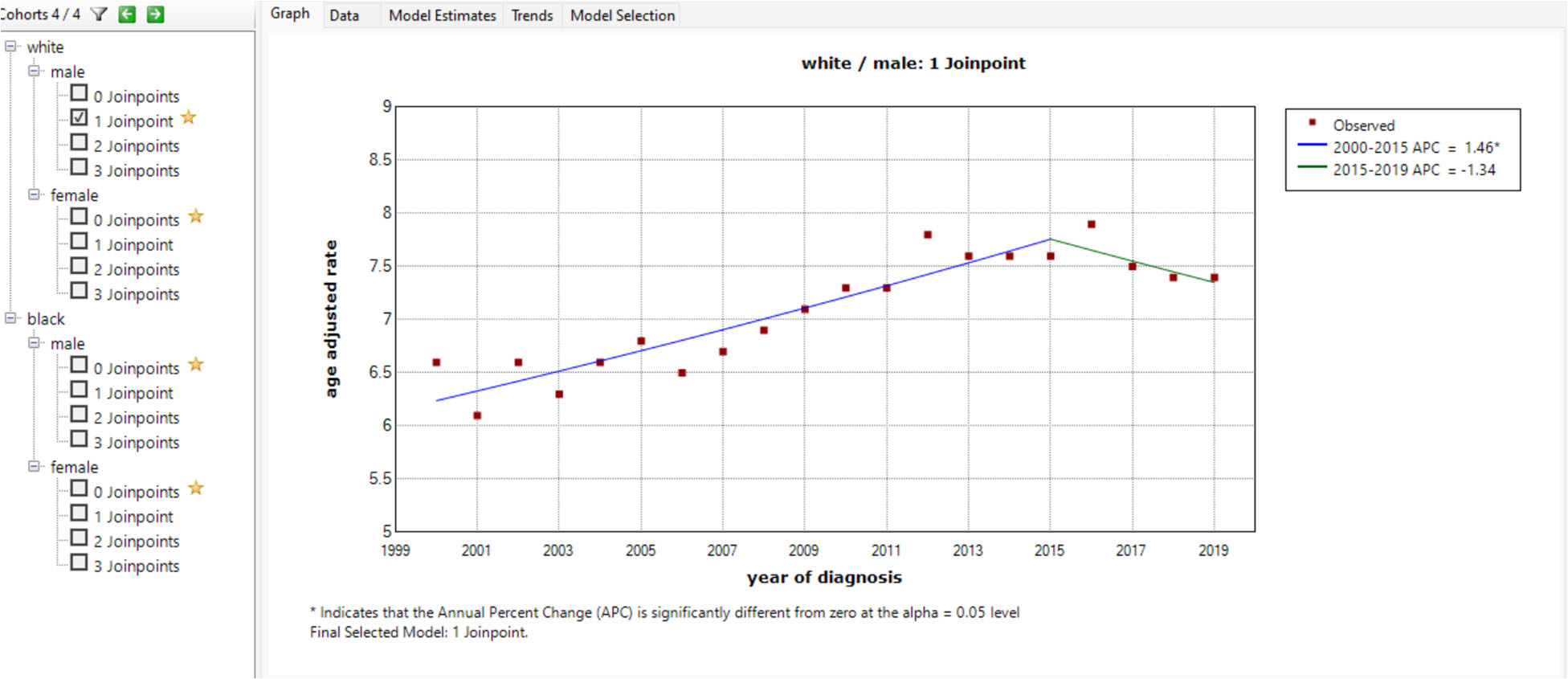
An increase in age-adjusted incidence rate APC of 1.46 between 2000 – 2015, and a decline in age adjusted incidence rate APC to 1.34 between 2015 – 2019.

The joinpoint analysis by sex and year (Figure 3) showed an increased in the age-adjusted incidence rate of 7.76 between 2000–2015, and a declined in the age-adjusted incidence rate to 7.35 between 2015–2019. The APC between 2000–2015 was 1.46%, which was statistically significant. The APC between 2015–2019 was −1.34%. The joinpoint in 2015 was found among white male patients.

**Figure 4.**
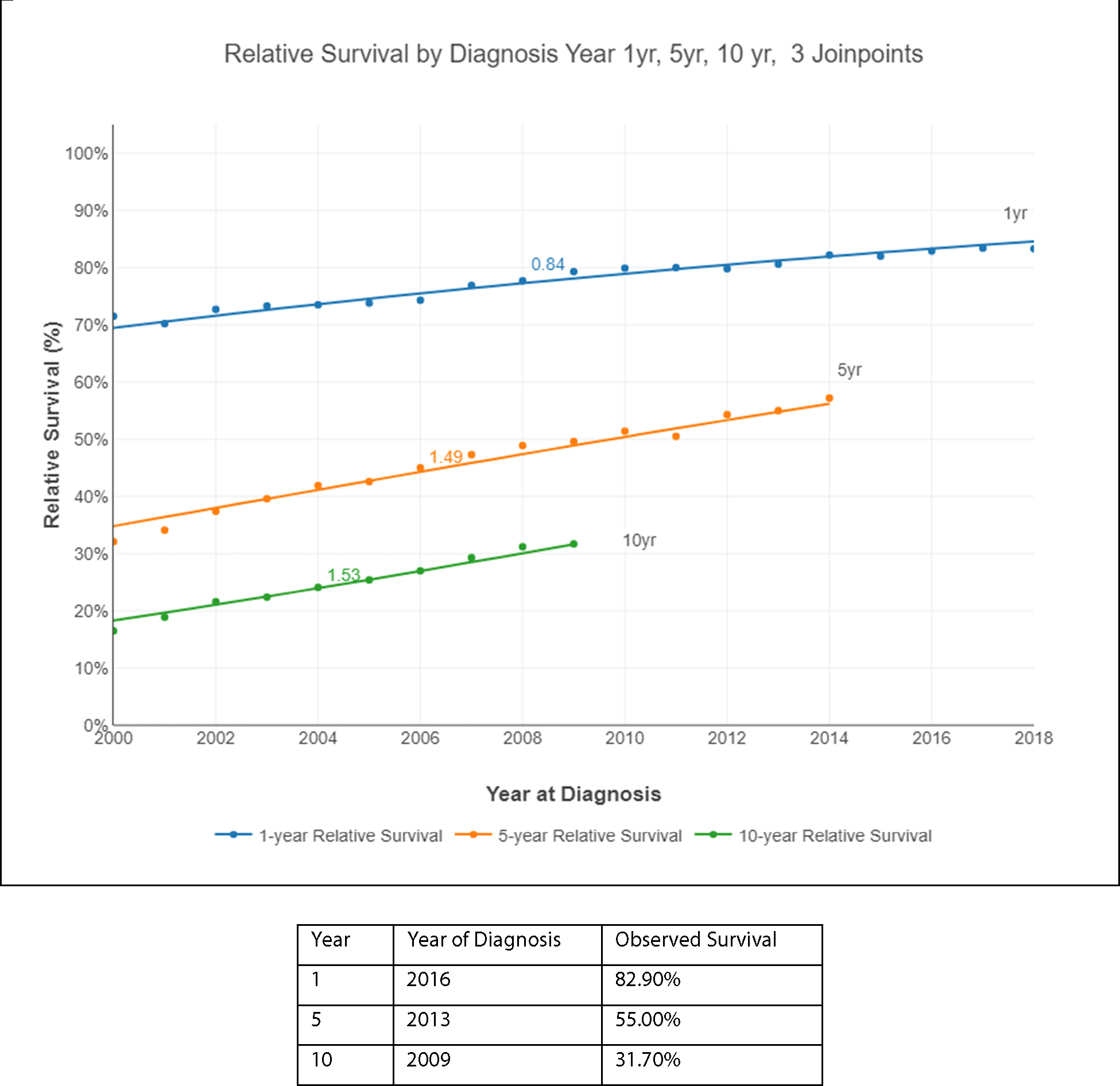
Relative Survival by diagnosis: Years 1, 5, and 10.

Figure 4 represents the Relative Survival (RS) by diagnosis for years 1, 5, and 10. Relative Survival for multiple myeloma patients has been increasing. For example, 1-year RS for patients with MM in 2000 was 71.5% compared with 82.2% in 2014 and 83.3% in 2018. The 5-year RS was 32.1% and increased significantly to 57.2% by 2014.

The largest decline in the death rates from multiple myeloma was the period between 2000–2008. The average absolute change in survival (AAC_S) at the 1-year interval in 2000 was 1.12%, the 5-year interval was 1.80%, and the 10-year interval was 1.68%, with statistical significance. Of note, the 5-year cancer survival rate in multiple myeloma increased an average of 1.8% for every year after diagnosis.

**Figure 5.**
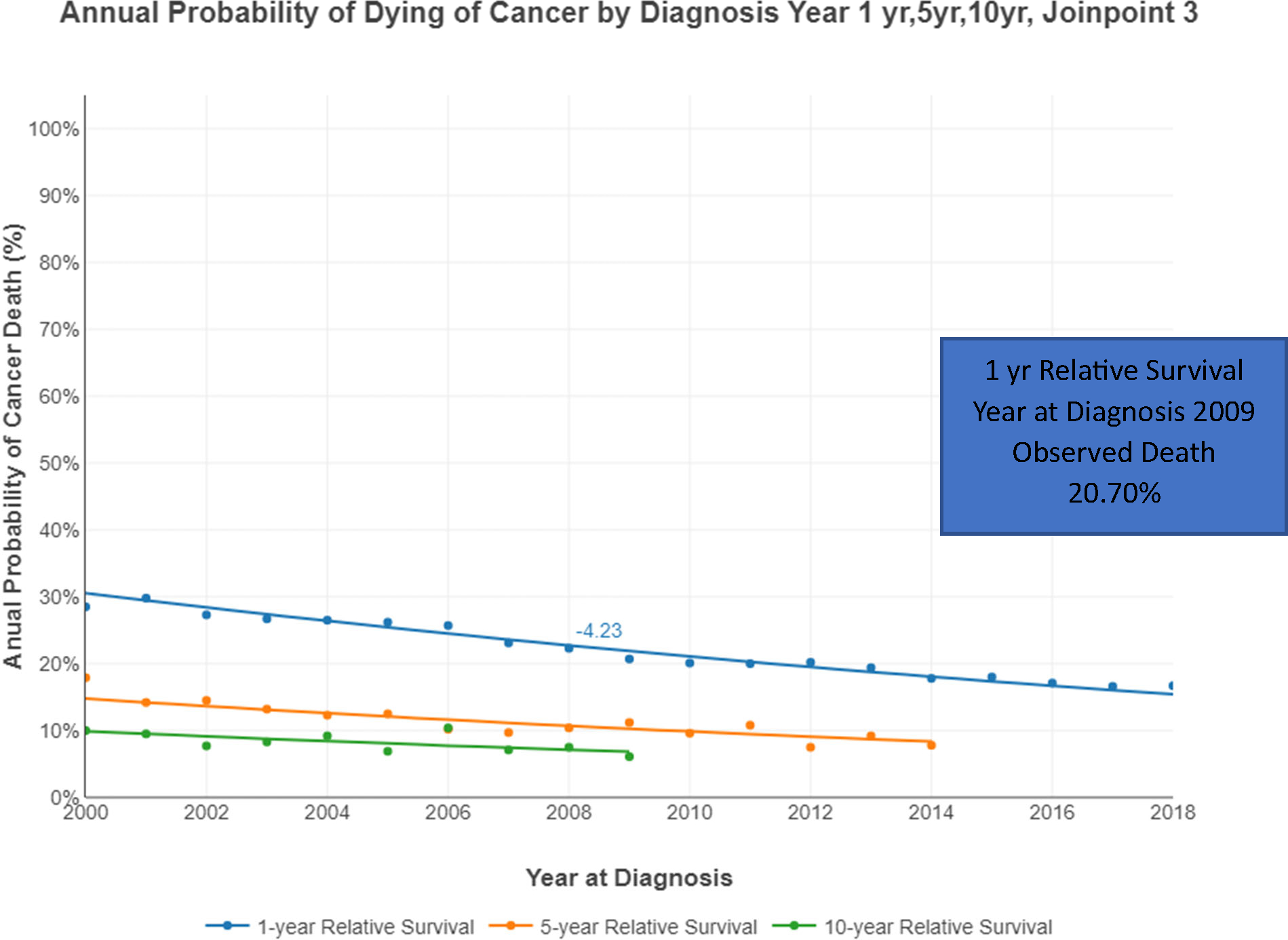
Annual Probability of Dying of Cancer by Diagnosis: Year 1, 5, 10 – 3 Joinpoints.

The annual probability of dying from multiple myeloma after 1, 5, and 10 years from diagnosis is represented in Figure 5. This probability decreased between 2000–2019. For example, the observed probability of death at the 1-year interval in 2000 was 28.5% but decreased to 16.7% in 2018. The 5-year interval probability decreased from 17.9% in 2000 to 7.8% in 2014. The 10-year interval probability also decreased from 10% in 2000 to 6.9% in 2009 (last year reported). The percentage change in the annual probability of dying from MM decreased in 2000 to −4.84%, in 2008 to −3.92 and in 2013 to −2.97 and it was statistically significant.

## Discussion

Cancer survival in patients with MM is increasing [13]. Our study showed that in 2000, the Relative Survival for patients with MM 1 year, 5 years, and 10 years after diagnosis was 71.5%, 32.1%, and 16.5% respectively. In contrast, the relative survival in 2014 for years 1 and 5 was 82.2% and 57.2% respectively. Our 5-year Relative Survival data is comparable to other studies from national registries in Europe. For example, in a study collecting data from 11 population-based cancer registries in Germany, the RS at 5 years increased from 39.9% in patients who were diagnosed from 2002–2004 to 47.9% in 2008–2010 [14–16]. A study using the Italian Modena Cancer registry also reported a 5-year RS rate of 45.7% for patients diagnosed with MM between 1988–1996, but increased to 49.9% during the years 1997–2005, and again to 55.7% during 2006–2009 [15]. Also, studies from New Zealand and the Netherlands included patients diagnosed with MM in the calendar years of 1990–2016 and 1989–2018, respectively, and showed a significant survival improvement in all regional health authorities (RHAs) (p<0.001 for all RHAs) [16].

The history of treatments used for MM explains this improvement in survival, dating back to the 1940s, using agents such as nitrogen mustard-based alkylating chemotherapies, anthracyclines, and glucocorticoids [17]. Even using these chemotherapy agents, life expectancy was approximately 20 months, and the prolonged administration of these agents was limited by toxicities such as chemotherapy-induced myelodysplastic syndrome and frequent infections (including with opportunistic pathogens) [17]. Autologous stem cell transplants (ASCTs) became a widely used treatment starting in the 1990s. Using high-dose melphalan for myeloablation followed by hematopoietic stem cell infusion, ASCT was shown in randomized clinical trials and later supported by meta-analyses to have a survival benefit for MM patients [18].

Novel agents in the late 1990s and early 2000s for the treatment of MM included proteasome inhibitors (PIs) and immunomodulatory agents (IMiDs) [19]. Bortezomib was a breakthrough for 1st line and relapsed MM treatment. Other PIs such as carfilzomib and ixazomib, with different physicochemical structures and methods of binding to the proteasome, were approved for clinical use at that time [20]. Thalidomide was the first IMiD clinically used in the treatment of MM; it can inhibit angiogenesis, induce apoptosis of established neo vasculature, and exert immunomodulatory and anti-inflammatory properties [21]. Lenalidomide, a derivative of thalidomide, demonstrated in a long-term follow up after using the combination of Revlimid/bortezomib and Decadron, an overall response rate (ORR) of 97.1% after induction treatment and increased to 98.5% following ASCT, with 89.9% of patients reaching a very good partial response (VGPR) or better and 33.3% obtaining a stringent complete response (CR) following ASCT at a median follow-up time of 67 months [22]. Another derivative of thalidomide named pomalidomide showed even more anti-inflammatory and antiangiogenic features with fewer toxicities [23].

This past decade has brought sweeping changes to MM treatment, particularly with the development of immunotherapy. One such example came from the MAIA clinical trial, where daratumumab (an anti-CD38 antibody) was used for patients who did not qualify for ASCT. This study showed a significant PFS benefit using daratumumab with lenalidomide/dexamethasone versus lenalidomide and dexamethasone alone in MM patients ineligible for ASCT. The median PFS was not reached with double therapy compared to 34.4 months with single treatment (HR, 0.53; 95% CI, 0.43–0.66; P<0.0001) [24]. Another study that introduced a different mechanism of action, the ELOQUENT-2 trial, compared the use of elotuzumab (which targets SLAMF7) in conjunction with both lenalidomide and dexamethasone as opposed to lenalidomide and dexamethasone alone. That trial showed a significant increase in median PFS of 19.4 months with the triple therapy compared to 14.9 months using lenalidomide and dexamethasone alone (HR, 0.70; 95% CI, 0.57–0.85; P<0.001) [25].

Most recently, the introduction of CAR-T cell therapy (such as idecabtagene vicleucel), has shown good responses in patients with refractory/relapsed MM, many of whom were pretreated with numerous regimens prior; MRD-negative status was obtained in 26% of patients who were treated, with a median PFS of 8.8 months (95% confidence interval, 5.6 to 11.6) [26]. Bispecific antibodies such as teclistimab are another class of novel therapies showing promise. Teclistimab targets CD3 (expressed on the T-cell surface) and BCMA (expressed on the myeloma cell surface) and is now given for those with refractory or relapsed MM with prior exposure to triple therapy (IMiD, PI, and anti-CD38). The studies in its development showed a high rate of profound and prolonged response [9].

Additionally, the IMWG has recommended monitoring MRD with the use of different methods including the clonotypic peptide approach and mass spectrometry. The clonotypic method uses an extremely sensitive analysis to detect M-protein values as low as 0.001 g/L, allowing it to be an accurate serum-based MRD method [27]. The method of mass spectrometry is also emerging as an accurate way to monitor serum proteins. Preliminary studies have shown that the limit of detection for matrix-assisted laser desorption/ionization time-of-flight (MALDI-TOF) MS is <10 mg/L [28].

There were limitations in our study, much of which was due to the inherent difficulties navigating the SEER database. The data lacked important information in terms of staging and genetic risk. In addition, we were unable to risk-stratify patients according to the revised-international staging systems (R-ISS) and their genetic risk. The therapeutic regimens were not reported, as well as drug dosage, radiation dose, and whether or not the patient received stem cell transplantation. Patients with MM have high heterogeneity that cannot be overlooked.

Further studies will be needed to evaluate responses and relative survival of myeloma patients treated in this new age of immunotherapy, monitoring with new methodologies for MRD, and tailing treatments to specific genomic biomarkers.

## Figures and Tables

**Table 1.** Characteristics of patients with Multiple Myeloma aged 30 and older.

Characteristic	Number of patients	Percent
Gender	111,328	
Male	61,421	55.17%
Female	49,908	44.82%
Race		
White	82,265	73.89%
Black	23,396	21.01%
Asian	5,134	4.61%
Other	533	0.48%
Age		
30–34	321	0.28%
35–39	867	0.77%
40–44	2,024	1.81%
45–49	4,121	3.70%
50–54	7,230	6.49%
55–59	10,892	9.78%
60–64	14,669	13.17%
65–69	17,437	15.66%
70–74	16,621	14.92%
75–79	14,904	13.38%
80–84	11,838	10.63%
85+	10,404	9.34%

**Table 2.** Race and gender in multiple myeloma.

Race	Male	Female
White	76.7%	72%
Black	17.20%	21.20%
American Indian/ Alaskan native	0.50%	0.70%
Asian or Pacific Islander	5.50%	6.00%

## Abbreviations

MM: Multiple Myeloma
MGUS: Monoclonal Gammopathy of Undetermined Significance
SMM: Smoldering Multiple Myeloma (SMM)
IMWG: International Myeloma Working Group
PFS: Progression Free Survival
OS: Overall Survival
VGPR: Very Good Partial Response
CR: Complete Response
FLC: Free Light Chain
MRD: Minimal Residual Disease
CART: Chimeric Antigen Receptor (CAR) T-cells
SEER: The Surveillance, Epidemiologic, and End Results
NCI: National Cancer Institute
APC: Annual Percentage Change
AAPC: Average Annual Percent Change
PIs: Proteosome Inhibitors
IMiDs: Immunomodulatory agents
ASCT: Autologous Stem Cell Transplantation
R-ISS: Risk-stratify patients according to the revised-international staging systems
BCMA: B cell Maturation Antigen
